# Prevalence of intestinal parasitic infections and associated risk factors among students at Dona Berber primary school, Bahir Dar, Ethiopia

**DOI:** 10.1186/s12879-017-2466-x

**Published:** 2017-05-23

**Authors:** Tamirat Hailegebriel

**Affiliations:** Department of Biology, College of Science, Bahir Dar University, P.O. Box 79, Bahir Dar, Ethiopia

**Keywords:** Intestinal parasite, School children, Risk factors

## Abstract

**Background:**

Intestinal parasitic infections are still one of the major health concerns in developing countries. Monitoring of intestinal parasitic infection and associated risk factors are essential for intervention strategies. Therefore, the aim of this study was to assess the prevalence of intestinal parasitic infection and associated risk factors among students at Dona Berber primary school, Bahir Dar, Ethiopia.

**Methods:**

School based cross-sectional study was conducted among students at Dona Berber primary school from October 2015 to June 2016. Three hundred fifty nine students were involved in the study by providing stool specimens and detailed personal information. Students were selected by stratified and systematic random sampling method. Fresh stool samples were collected from each student and processed by formal-ether fecal concentration technique. Data were analyzed using SPSS version 20.0 statistical software and *p* value <0.05 were used as statistically significant.

**Results:**

Among the 359 students participated in the study, 235 (65.5%) were infected by one or more intestinal parasites. The rates of single and double parasitic infections among students were 49.6% and 16.2%, respectively. The most prevalent parasite detected in the study was *E. histolytica/dispar* (24.5%) followed by hookworm (22.8%). Among the different variables assessed in the study, family size of 6 (AOR = 4.90; 95% CI, 2.03–11.83), irregularly shoe wearing habit (AOR = 2.85; 95% CI, 1.53–5.32) and unclean finger nail (AOR = 3.68; 95% CI, 1.87–7.26) were independently predict intestinal parasitic infections. Student drinking well water (AOR = 2.51; 95% CI, 2.30–4.86) and unclean finger nail (AOR = 4.42; 95% CI, 2.55–7.65) were strongly associated with *E. histolytica/dispar* infection. Likewise, irregular shoe wearing habit (AOR = 14.13; 95% CI, 7.06–28.29) was strongly associated with hookworm infections.

**Conclusion:**

High prevalence of intestinal parasitic infection among the study participants demands improvement of health education, environmental sanitation and quality of water sources.

## Background

Intestinal parasitic infection is one of the major health problems globally and up to 3.5 billion people are infected and around 450 million people are ill due to intestinal parasites [1]. The majority of these intestinal parasitic infections are concentrated in developing countries. The problem are more serious in Sub-Saharan Africa, Asia and Latin America associated with inadequate water supply, environmental sanitation, fast population growth, and other economic and social and problems [2]**.**

Intestinal parasitic infections are more prevalent among children as compared with the general population. About 12% of the global disease burdens caused by intestinal parasites is observed among children with age ranges from 5 to 14 years in developing countries [3]. Up to 270 million preschool and 600 million school children are living in area where high transmission of parasitic worm [4]. These indicated that children are the major risk group for parasitic infection in many developing countries.

Protozoa and helminthic parasites are the known parasites that affect the gastrointestinal cavity. Intestinal parasites such as *Ascaris lumbricoides, Trichuris trichiura* and *hookworm* are the most prevalent and affect about one-sixth of the world population [5]. *A. lumbricoides* is responsible for about 1.2 billion infections globally while *T. trichiura* and *hookworm* infection accounts about 795 million and 740 million, respectively [6]**.** Among the protozoan parasite, *E. histolytica* and *Giardia lamblia* are the most dominant cause of intestinal morbidity in children**.**

High prevalence of intestinal parasitic infection were reported among school children in Sub Saharan African countries including 27.7% to 95% [7–14] in Ethiopia, 90% in Central Sudan [15], 50.0% in Rwanda [16], 48.7% in Tanzania [17] and 84.7% in Burkina Faso [18]. High prevalence of intestinal parasitic infection in the region is associated with personal hygiene, socio economic status and educational level of the community.

The presence of intestinal parasitic infections may have multiple effects among children including physical and mental developments. The presence of chronic and heavy intestinal parasitic infection cause intestinal bleeding, malabsorption of nutrients, nutritional deficiency, destruction of cells and tissues and other associated effects. The overall effect of these results in growth retardation, reduced mental development, school absenteeism, low academic performance, susceptible to malnutrition and infection [19].

Several studies indicate the problem and severity of intestinal parasitic infection in Ethiopia. In line with this, continuous monitoring of intestinal parasitic infection and their associated risk factors are essential among school children in the country. There is no recent information about intestinal parasitic infection and associated risk factors in the study area. Therefore, the present study was aim to determine the prevalence of intestinal parasitic infection and associated risk factors among school children at Dona Berber primary school, Bahir Dar, Ethiopia.

## Methods

### Study design and study area

A school based cross-sectional study was conducted from October 2015 to June 2016 among students at Dona Berber primary school, Bahir Dar, Ethiopia. The school was located at the southern part of Bahir Dar city. Bahir Dar is located at 11^o^6’N, 37^o^38’E and about 578 km far from the capital city of the country, Addis Ababa. Bahir Dar is one of the fast growing cities in the country and serves as capital city of Amhara regional state. According to 2007 national population census, the city serves as home for about 180,094 people, of which majority of them are children [20].

### Study population and sample size determination

The population of the study was students attending at Dona Berber primary school from grade 1 to grade 4. The total numbers of students from grade 1 to grade 4 were 1274 in the study period. A total of 19 classes in the school and each class contain an average of 67 students. The target students were selected by stratified sampling based on their educational level (from grade 1 to grade 4) and quota was allocated to each grade based on the total number of students. The actual numbers of students participated in the study from each class were selected by systematic random sampling using class roster as reference.

The sample size was determined by statistical formula n = Z^2^xP (1-P)/d^2^ where P (prevalence of intestinal parasite in the area), d (at 5% marginal error) and standard score (Z) at 95% confidence interval. The prevalence of intestinal parasitic infection in Bahir Dar was reported as 41% [21]**.** The sample size determined for the study was 372. In order to minimize errors caused by dropout, the sample size were increased by 5%. The final sample size used in the study was 390.

### Sample collection and processing

Structured questionnaire were prepared to gather relevant information from each student. Selected students for the study were interviewed to obtain information about age, grade, sex, religion, water sources, toilet availability, family education, family income, shoe wearing and hand washing habit. As the same time, the finger nail cleanness of each student was checked by data collectors. After collecting the information, all students were asked to bring fecal specimens using properly labeled stool cap with an applicator stick. About 2 g of stool specimens were collected from each student and mixed with 10% formalin for preservation.

The preserved fecal specimens were transported to the laboratory of Biology Department, Bahir Dar University. All specimens were processed using formal-ether fecal concentration techniques as indicated in WHO guidelines [22] by trained laboratory technologist. All developmental stages of parasite (cyst, egg, larvae, adult worm and worm segment) were recorded.

### Quality control

Among the total positive samples, 10% were selected randomly for re-examination. The selected samples were processed and examined by experienced laboratory technologist who did not have information about the previous result. The results of the new laboratory examination were used as a quality control.

### Data analysis

Statistical package for social science (SPSS) version 20.0 were used to analyze the collected data. The associations of potential risk factors and presence of intestinal parasitic infection were performed by binary logistic regression analysis model. The results of the association were considered as significant when the *p* value was below 0.05.

## Results

### Socio-demographic characteristic of study participants

A total of 390 students were selected for this study. Among them, 31 (7.9%) students were excluded from the analysis due to either incomplete information or insufficient fecal specimen production. As a result, 359 primary school children were involved in the study. Among these students, 182 (50.7%) were female and the remaining 177 (49.3%) male. The students participated in the study were age ranges from 7 to 14 years and from grade 1 to grade 4 (Table 1). The average age of the students involved in the study was 10.1 (SD ± 2.2) years.

**Table 1:** Sociodemographic characteristic of school children and their family at Dona Berber first cycle primary school, Bahir Dar, Ethiopia, 2015/2016

Variables	Categories	Frequency	Percentage
Sex student	Female	182	50.7
	Male	177	49.3
Age of students	7–8	102	28.4
	9–10	113	31.5
	11–12	87	24.2
	13–14	57	15.9
Grade students	1	102	28.4
	2	93	25.9
	3	85	23.7
	4	79	22
Family size	>6	97	27
	6	123	34.3
	5	96	26.7
	<5	43	12
Family monthly income (ETB^a^)	< 500	123	34.3
	500–2000	215	59.9
	>2000	21	5.8
Father education	Illiterate	97	27
	Primary school	169	47.1
	Secondary school	43	12
	Diploma and above	50	13.9
Mother education	Illiterate	177	49.3
	Primary school	138	38.4
	Secondary school	25	7
	Diploma and above	19	5.3
Water source	Well water	63	17.5
	River	25	7
	Pipe water	271	75.5
Hand washing habit	Sometimes	146	40.7
	Always	213	59.3
Shoe wearing habit	Not at all	20	5.6
	Sometimes	134	37.3
	Always	205	57.1
Religion	Muslim	9	2.5
	Orthodox	337	93.9
	Protestant	13	3.6
Finger cleanness	Not clean	125	34.8
	Clean	234	65.2
Toilet availability	Not available	67	18.7
	Available	292	81.3

^a^Ethiopian birr

### Prevalence of intestinal parasites among study participants

Among the 359 students participated in the study, 235 (65.5%) were infected by one or more parasitic organism (Table 2). The prevalence of intestinal parasitic infection was 32% and 33.5% among female and male students, respectively. The rate of single, double and triple parasitic infections were 174 (48.5%), 58 (16.2%) and 3(0.8%), respectively (Table 2). Nine intestinal parasitic organisms were recorded from the study participants. Among these intestinal parasites, *E. histolytica/dispar* was the most prevalent 88 (24.5%) followed by *hookworm* 82 (22%), *A. lumbricoides* 49 (13.6%) and *G. lamblia* 41(11.4%) (Table 2).

**Table 2:** Prevalence of parasitic infection among children by sex at Dona Berber first cycle primary school in Bahir Dar, Ethiopia, 2015/2016

Parasite species		Parasite infected students by sex			
		Female No. (%)	Male No. (%)	Total No. (%)	Chi-Square *P*- value
Protozoa	*E. histolytica*	48 (13.4)	40 (11.1)	88 (24.5)	0.41
	*G. lamblia*	18 (5)	23 (6.4)	41 (11.4)	0.36
	*E. vermicularis*	0 (0)	1 (0.3)	1 (0.3)	0.31
	*T. hominins*	3 (0.8)	2 (0.6)	5 (1.4)	0.68
Helminths	*Hookworm*	39 (10.9)	43 (12)	82 (22.8)	0.52
	*A. lumbricoides*	21 (5.8)	28 (7.8)	49 (13.6)	0.24
	*H. nana*	9 (2.5)	8 (2.2)	17 (4.7)	0.85
	*S. stericoralis*	6 (1.7)	10 (2.8)	16 (4.5)	0.28
	*T. trichiura*	5 (1.4)	5 (1.4)	10 (2.8)	0.96
Single infection		84 (23.4)	90 (25.1)	174 (48.5)	0.37
Double infection		29 (8.1)	29 (8.1)	58 (16.2)	0.91
Triple infection		2 (0.6)	1 (0.3)	3 (0.8)	0.58
Over all infection		115 (32)	120 (33.4)	235 (65.5)	0.34

### Potential risk factors associated with intestinal parasites infection

Univariate logistic regression analyses model showed that family size, drinking water sources, shoe wearing habit and fingernail cleanness were major risk factors for intestinal parasitic infection. Multivariate logistic regression analysis showed that student do not always wash their hand before meal (AOR = 2.33; 95% CI, 1.29–4.19), drinking of well water (AOR = 2.84; 95% CI, 1.09–7.45), unclean finger nail (AOR = 3.68; 95% CI, 1.87–7.26) and irregular shoe wearing habits (AOR = 2.85; 95% CI, 1.53–5.32) were independently predict intestinal parasitic infections (Table 3). Likewise, student belonging larger family size were at higher risk of infection by parasitic organism as compared with students with small family size (Table 3). The odd of being infected by parasitic infection were increased by 5 fold among students belonging to a family size of 6 as compared with small family size. The present study showed that there were no statistical significant association of intestinal parasitic infection with sex, grade, religion and age of students involved in the study (*p* > 0.05).

**Table 3:** Bivariate and multivariate logistic regression analysis of potential risk factors associated with parasitic infection among children at Dona Berber first cycle primary school in Bahir Dar, Ethiopia, 2015/2016

Risk factors	Categories	Parasitic infection			Crude OR (CI 95%)	Adjusted OR (CI 95%)^#^
		Positive No (%)	Negative No (%)	Total No (%)		
Family size	Above 6	77 (79.4)	20 (20.6)	97 (27)	5.89 (2.69–12.9)**	3.62 (1.37–9.59)*
	6	90 (73.2)	33 (26.8)	123 (34.3)	4.17 (2.01–8.66)**	4.90 (2.03–11.83)**
	5	51 (53.1)	45 (46.9)	96 (26.7)	1.73 (0.83–3.60)	2.79 (1.14–6.85)*
	Less than 5	17 (39.5))	26 (60.5)	43 (12)	1.00	1.0
Family monthly income (ETB^@^)	< 500	102 (82.9)	21 (17.1)	123 (34.3)	6.48 (2.42–17.3)**	1.94 (0.59–6.34)
	500–2000	124 (57.7)	91 (42.3)	215 (59.9)	1.82 (0.74–4.49)	1.70 (0.59–4.93)
	>2000	9 (42.9)	12 (57.1)	21 (5.8)	1.00	1.00
Father education	Illiterate	81 (83.5)	16 (16.5)	97 (27)	9.00 (4.09–19.79)**	1.67 (0.52–5.39)
	Primary school	109 (64.5)	60 (35.5)	169 (47.1)	3.23 (1.67–6.24)**	1.68 (0.66–4.25)
	Secondary school	27 (62.8)	16 (37.2)	43 (12)	3.00 (1.29–6.99)*	1.23 (0.41–3.69)
	Diploma and above	18 (36)	32 (64)	50 (13.9)	1.00	1.00
Mother education	Illiterate	137 (77.4)	40 (22.6)	177 (49.3)	9.59 (3.26–28.24)**	1.88 (0.45–7.84)
	Primary school	80 (58)	58 (42)	138 (38.4)	3.86 (1.32–11.32)*	1.23 (0.32–4.77)
	Secondary school	13 (52)	12 (48)	25 (7)	3.03 (0.84–10.99)	1.46 (0.33–6.50)
	Diploma and above	5 (26.3)	14 (73.7)	19 (5.3)	1.00	1.00
Drinking water source	Well water	56 (88.9)	7 (11.1)	63 (17.6)	5.64 (2.48–12.82)**	2.84 (1.09–7.45)*
	River	20 (80)	5 (20)	25 (7)	2.82 (1.03–7.73)	1.00 (0.30–3.36)
	Tape water	159 (58.7)	112 (41.3)	271 (75.5)	1.00	1.00
Hand washing habit	Sometimes	118 (80.8)	28 (19.2)	146 (40.7)	3.46 (2.11–5.66)**	2.33 (1.29–4.19)**
	Always	117 (54.9)	96 (45.1)	213 (59.3)	1.00	1.00
Shoe wearing habit	Not at all	19 (95)	1 (5)	20 (5.6)	16.73 (2.20–127.4)*	7.80 (0.88–69.39)
	Sometimes	107 (79.9)	27 (20.1)	134 (37.3)	3.49 (2.11–5.77)**	2.85 (1.53–5.32)**
	Always	109 (53.2)	9646.8)	205 (57.1)	1.00	1.00
Fingernail cleanness	Not clean	108 (86.4)	17 (13.6)	125 (34.8)	5.35 (3.02–9.49)**	3.68 (1.87–7.26)**
	Clean	127 (54.3)	107 (45.7)	234 (65.2)	1.00	1.00
Toilet availability	Not available	60 (89.6)	7 (10.4)	67 (18.7)	0.17 (0.07–0.40)**	0.74 (0.27–2.04)
	Available	175 (59.9)	117 (40.1	292 (81.3)	1.00	1.00
Sex of students	Female	115 (63.2)	67 (36.8)	182 (50.7)	0.82 (0.53–1.26)	-
	Male	120 (67.8)	57 (32.2)	177 (49.3)	1.00	
Age (years)	7–8	70 (68.6)	32 (31.4)	102 (28.4)	1.09 (0.55–2.18)	-
	9–10	74 (65.5)	39 (34.5)	113 (31.5)	0.95 (0.48–1.86)	-
	11–12	53 (60.9)	34 (39.1)	87 (24.2)	0.78 (0.39–1.57)	-
	13–14	38 (66.7)	19 (33.3)	57 (15.9)	1.00	
Grade	1	70 (68.6)	32 (31.4)	102 (28.4)	1.49 (0.81–2.75)	-
	2	61 (65.6)	32 (34.4)	93 (25.9)	1.29 (0.70–2.41)	-
	3	57 (67.1)	28 (32.9)	85 (23.7)	1.39 (0.73–2.62)	-
	4	47 (59.5)	32 (40.5)	79 (22)	1.00	
Religion	Muslim	5 (55.6)	4 (44.4)	9 (2.5)	0.78 (0.14–4.39)	-
	Orthodox	222 (65.9)	115 (34.1)	337 (93.9)	1.21 (0.39–3.77)	-
	Protestant	8 (61.5)	5 (38.5)	13 (3.6)	1.00	

Not: *= *p* < 0.05, **= *p* < 0.01, ^#^= adjusted (multivariate regression model) for family size, family income, drinking water source, shoe wearing habit, finger nail cleanness, hand washing habit before meal, toilet availability, mother and father education, ^@^= Ethiopian birr.

### Risk factors associated with *E. histolytica/dispar* and hookworm infection

*Entamoeba histolytica/dispar* and hookworm were the most frequent intestinal parasites detected from the study participants (Table 2). Univariate logistic regression analysis showed that hand washing habit before meal, finger nail cleanness, and drinking water source were risk factors for *E. histolytica/dispar* infection. Drinking of well water (AOR = 2.51; 95% CI, 2.30–4.86), unclean finger nail (AOR = 4.42; 95% CI, 2.55–7.65) and irregular hand washing habit before meal (AOR = 2.32; 95% CI, 1.56–3.97) were predict infections of *E. histolytica/dispar* (Table 4). Multivariate logistic regression analysis indicated that shoe wearing habit was the independent risk factor for hookworm infection. Absence of shoe (AOR = 10.92; 95% CI, 3.72–32.06) and irregularly use of protective shoe (AOR = 14.13; 95% CI, 7.06–28.29) were independently predict hookworm infection (Table 4).

**Table 4:** Binary logistic regression analysis of potential risk factors associated with *E. histolytica/dispar* and hookworm infection among children at Dona Berber first cycle primary school in Bahir Dar, Ethiopia, 2015/2016

Dependent variables	Categories	E. histolytica/dispar			Crude OR (CI 95%)	Adjusted OR (CI 95%) #
		Positive No (%)	Negative No (%)	Total No (%)		
Water source	Well water	28 (44.4)	35 (55.6)	63 (17.5)	3.07 (1.72–5.47)**	2.51 (2.30–4.86)**
	River	4 (16)	21 (84)	25 (7)	0.73 (0.24–2.22)	0.36 (0.11–1.19)
	Tape water	56 (20.7)	215 (79.3)	271 (75.5	1.00	1.00
Hand washing habit	Sometimes	53 (36.3)	93 (63.7)	146 (40.7)	2.99 (1.77–4.76)**	2.32 (1.56–3.97)**
	Always	35 (16.4)	178 (83.6)	213 (59.3)	1.00	1.00
Fingernail cleanness	Not clean	55 (44)	70 (56)	115 (34.8)	4.79 (2.87–7.97)**	4.42 (2.55–7.65)**
	Clean	33 (14.1)	201 (85.9)	234 (65.2)	1.00	1.00
Toilet availability	Not available	25 (37.3)	42 (62.7)	67 (18.7)	0.46 (0.26–3.82)*	0.88 (0.45–1.74)
	Available	63 (21.6)	229 (78.4)	292 (81.3)	1.00	1.00
		Hook worm				
		Positive No (%)	Negative No (%)	Total No (%)		
Shoe wearing habit	Not at all	8 (40)	12 (60)	20 (5.6)	10.72 (3.7–31.2)**	10.92 (3.7–32.1)**
	Sometimes	62 (46.3)	72 (53.7)	134 (37.3)	13.85 (7.1–27.2)**	14.13 (7.1–28.3)**
	Always	12 (5.9)	193 (94.1)	205 (57.1)	1.00	1.00
Toilet availability	Not available	23 (34.3)	44 (65.7)	67 (18.7)	0.48 (0.27–0.87)*	1.09 (0.57–2.08)
	Available	59 (20.2)	233 (79.8)	292 (81.3)	1.00	1.00

Not: *= *p* < 0.05, **= *p* < 0.01, #= adjusted (multivariate regression model) for drinking water source, finger nail cleanness, hand washing habit before meal and toilet availability for *E. histolytic/dispar* and shoe wearing habit and toilet availability for hookworm infection

## Discussion

Epidemiological studies on the prevalence of intestinal parasitic infection and predisposing risk factors in school children are essential to design appropriate intervention strategies. The present study was therefore aim to assess the prevalence of intestinal parasitic infection and associated risk factors in school children. The overall prevalence of intestinal parasitic infection in the present study was 65.5%, which is comparable with 65% -71% reported from different parts of Ethiopia [23, 24] and Morocco [25]. The present finding was a bit higher in comparison with 26.2% -54.5% reported in different parts of Ethiopia [8, 26, 27] and those of 50% [16], 48.7% [17], 30% [28] and 42.3% [29] reported from Rwanda, Tanzania, Sudan and Iran, respectively. In contrast to our finding, high prevalence of intestinal parasitic infections were reported from Ethiopia (71.8% - 95%) [12–14, 30, 31], Central Sudan (90.4%) [15], Burkina Faso (84.7%) [18] and Yemen (90%) [32]. These reported differences in prevalence of intestinal parasites among different studies might be associated with difference in parasitological methods used, level of environmental sanitation, drinking water source, family education and personal hygiene. High prevalence of intestinal parasitic infection is a direct manifestation of poor environmental sanitation and low level of awareness.

The most prevalent intestinal parasite in the present study was *E. histolytica/dispar* (24.5%), which is in agreement with 21.6% - 27.3% reported from school children elsewhere [13, 14]. The present finding revealed high prevalence of *E. histolytica/dispar* in comparison with 8.2% -17.1% reported from different parts of Ethiopia [24, 30, 33], 15.5% in Sudan [28] and 8.2% in Saudi Arabia [34]. In contrast to our finding, high prevalence *E. histolytica/dispar* were reported from Rwanda (54.5%) [16], Burkina Faso (66.5%) [18] and Yemen (64%) [32]. The difference in prevalence of *E. histolytica/dispar* among different studies might be explained by difference in level of contamination of drinking water source, consumption of raw vegetables and hand washing habits of the study participants. Hand washing habit among study participants, consumption of raw vegetables and level of environmental contamination are varied from locality to locality that results in difference in prevalence of amoebic infection among studies.

Hookworm was the second most prevalence parasitic infection among students in the study, which is in agreement with 19% - 22.8% reported elsewhere [13, 31, 35]. However, the prevalence of hookworm infection in the present study was lower than other studies reported from Ethiopia [24, 33] and Western Cote D’Ivoire [36]. These variations in prevalence of hookworm infection among studies could be attributed to difference in environmental sanitation, shoe wearing habit of the students and differ in parasitological methods used during the study. High prevalence of hookworm infections among children was generally reported from rural areas with absence of protective shoe.

The present finding showed that *E. histolytica/dispar* infection was strongly associated with drinking water source, hand washing habit and unclean finger nail. In line with the present finding, the association of *E. histolytica/dispar* with drinking water source and hand washing habit before meal was reported elsewhere [37, 38]. Contamination of drinking water source, poor personal hygiene and lack of regular hand washing habits are major contributing factors for high *E. histolytica/dispar* infection*.*

The most important determinant of hookworm infection in the present study was shoe wearing habit of the study participants. The likely hood of being infected by hookworm was increased more than 10 folds among students who did not use protective shoe as compared with students regularly use protective shoe. The association of hookworm infection and shoe wearing habit is supported by several studies reported elsewhere [7, 9, 31, 39]. Lack of regular shoe wearing habit is known to be the major contributing factor that leads to high hookworm infection. This finding indirectly indicates the contamination of locality and playground of students with human fecal matters.

Polyparasitism is one of the common problems in developing countries. The prevalence of multiple parasitic infections in the present study was about 17%, which is in agreement with 18.5% [40] and 18.4% [24] reported from Central Sudan and Motta Town, respectively. However, our finding is lower than other reports from Ethiopia (28.5%) [13] and Burkina Faso (53.5%) [18]. The difference in prevalence of multiple parasitic infections at a time might be varied in relation to level of environmental contamination, level of awareness about parasitic infection and socioeconomic factors. This finding suggests that there might be low level of knowledge, attitude and practice towards intestinal parasitic infection in the study area.

The present study showed that family size, drinking water source, hand washing habit of before meal, shoe wearing habit and finger nail cleanness were strongly associated with the presence of intestinal parasitic infection. Similar association of intestinal parasitic infection with the aforementioned variables was reported from Ethiopia [24] and Saudi Arabia [41]. Hands and fingers of students might be easily contaminated with soil that contains cyst and eggs of parasitic organism that leads to intestinal infection.

The present study showed that family size was strongly associated with intestinal parasitic infection. The likely hood of being infected by intestinal parasites was increased by about 5 folds among students belonging in a family size of above 5 as compared with students belonging in a small family size. The present finding is in agreement with other studies conducted in Ethiopia [8, 27, 42], Iran [43] and Saudi Arabia [41]. The possible explanation of this association might be related to personal hygiene, environmental sanitation and other nutritional related problems. As a family size increase, there might be a problem of overcrowding, undernutrition, poor sanitation and personal hygiene within the family that create ideal condition for parasitic transmission as well as increase susceptibility of family members to parasitic infections.

The risk of being infected by intestinal parasitic infection were increased by 2 folds among students who do not frequently wash their hand before meal as compared with students regularly washed their hand before meal. Similar association of intestinal parasites and hand washing habit of students were reported from Ethiopia [13, 24, 31, 44] and Saudi Arabia [41]. Likewise, the likely hood of being infected by intestinal parasites were increased by nearly 3 folds among students do not frequently used protective shoe as compared with student regularly used protective shoe. Similar findings of association between intestinal parasitic infection and shoe wearing habit of students were reported from elsewhere [13, 24, 26, 42]. The possible explanations of association of shoe wearing habit and increase parasitic infection might be due to increase chance of hookworm and *S. stercoralis* infection. Lack of protective shoe is known risk factors to increase the chance of hookworm and *S. stercoralis* infection.

Finger cleanness and source of drinking water were strongly associated with presence of intestinal parasites. The risks of being infected by intestinal parasites were increased by 3.68 folds among students with unclean finger nail as compared with students who have clean finger nail. Similar association of intestinal parasitic infection with unclean finger nail were reported in different parts of Ethiopia [13, 27, 42, 44] and Saudi Arabia [41]. Similarly, the likely hood of being infected by intestinal parasites was increased by nearly 3 folds among students who drink well water as compared with students who drink tape water. The present finding of association of intestinal parasite with drinking water is supported by other results reported elsewhere [14, 24, 31].

## Conclusions

The present study revealed that there is high prevalence of intestinal parasitic infection among student in the target primary school. Among the different potential risk factors assessed in the study larger family size, poor hand washing habit, unclean finger nail, drinking water source and absence of protective shoe were strongly associated with intestinal parasitic infection. Therefore, all stakeholders should give attention to raise awareness about control of intestinal parasitic infection, personal and environmental hygiene, and improving the quality of drinking water source.

## Abbreviations

AOR: Adjusted odd ratio
CI: Confidence interval
COR: Crude odd ratio
SPSS: Statistical package for social science
WHO: World health organization
